# Evolution of Quality Management in Italian Healthcare (2016-2024) Beyond Mandatory Accreditation Through the International Organization for Standardization (ISO), Joint Commission International (JCI), and the New ISO 7101 Standard: A Scoping Review

**DOI:** 10.7759/cureus.107579

**Published:** 2026-04-23

**Authors:** Giuseppe Fumai, Claudio Morelli, Francesco Menolascina, Annamaria Fontana, Gianluca Laricchia

**Affiliations:** 1 Department of Orthopedic Rehabilitation, University of Foggia, Foggia, ITA; 2 Department of Orthopedic Rehabilitation, Rehcura Rehabilitation Facility, Adelfia, ITA; 3 Department of Oncology, Scientific Institute for Research, Hospitalization and Healthcare (IRCCS), Giovanni Paolo II Cancer Institute, Bari, ITA; 4 Department of Radiology, University of Bari Aldo Moro, Bari, ITA; 5 Legal Department, Bari Bar Association, Bari, ITA; 6 Department of Gynecology and Obstetrics, University of Foggia, Foggia, ITA

**Keywords:** healthcare quality management, institutional accreditation, iso 7101, iso 9001, italy, jci, risk management, scoping review

## Abstract

In the Italian National Health Service (Servizio Sanitario Nazionale, SSN), healthcare quality governance operates through a dual-track system: mandatory institutional accreditation regulated at the regional level under Legislative Decree 502/1992, and voluntary third-party certifications such as International Organization for Standardization (ISO) 9001:2015 and Joint Commission International (JCI). The enactment of Law 24/2017 ("Gelli-Bianco") mandated clinical risk management (CRM), while ISO 7101:2023 introduces a paradigm shift toward people-centered quality management.

This scoping review maps the adoption and integration of these quality frameworks in Italian healthcare. The review was conducted following the updated JBI methodology and reported in accordance with the Preferred Reporting Items for Systematic Reviews and Meta-Analyses Extension for Scoping Reviews (PRISMA-ScR) checklist. The protocol was prospectively registered on the Open Science Framework (OSF). A systematic search was performed in PubMed/MEDLINE and Scopus from January 2016 to December 2024, complemented by a targeted grey literature search. The Population, Concept, Context (PCC) framework guided the research question. Data were extracted into a predefined charting table and synthesized narratively.

Twenty-six sources were included. The Italian accreditation system shows persistent regional heterogeneity, with no peer-reviewed studies evaluating accreditation effectiveness during the review period. Law 24/2017 remains incompletely implemented, with 72% of clinical risk managers (CRMs) lacking regular interaction with regional centers. ISO 9001:2015 is the most widely studied voluntary standard, with 73.3% of respondents reporting continuous improvement. JCI accreditation remains concentrated in approximately 28 highly specialized centers. The first empirical study on ISO 7101:2023 demonstrated significant improvements in patient health literacy (66.35 to 76.29, p<0.001). The Agenzia Nazionale per i Servizi Sanitari Regionali (AGENAS) Programma Nazionale Esiti (PNE) documents a persistent North-South quality divide.

The Italian healthcare quality landscape is evolving from a compliance-driven model toward an integrated, multi-framework ecosystem. ISO 7101:2023 represents a strategic inflection point as a potential unifying paradigm. Future research should prioritize the empirical evaluation of ISO 7101 adoption in Italian settings and the investigation of the Southern quality governance gap.

## Introduction and background

Healthcare quality assurance has evolved from a purely clinical concern into a multidimensional governance challenge encompassing organizational performance, patient safety, and regulatory compliance [1]. In European healthcare systems, two distinct yet complementary approaches to quality assurance have historically coexisted: mandatory accreditation programs, typically administered by public authorities, and voluntary certification or accreditation through independent bodies [2]. The interplay between these mechanisms shapes the quality landscape of each national health system, influencing clinical governance, resource allocation, and patient outcomes.

In Italy, the Servizio Sanitario Nazionale (SSN) established institutional accreditation (accreditamento istituzionale) through Legislative Decree 502/1992, creating a three-tier system of authorization, accreditation, and contractual agreements that governs the relationship between healthcare providers and the public health service [3,4]. This framework delegates accreditation authority to Italy’s 20 regional governments, resulting in a mosaic of regional requirements that, despite a common legislative foundation, differ substantially in their implementation, stringency, and quality dimensions assessed [3]. The accreditation system was further refined by Legislative Decree 229/1999, which shifted from a liberalized market approach to a more regulated system aligned with regional health planning needs.

A pivotal development occurred with the enactment of Law 24/2017, commonly known as the “Gelli-Bianco Law,” which recognized patient safety as a fundamental right within the Italian legal framework [5,6]. This legislation mandated the establishment of clinical risk management (CRM) centers in all Italian regions and the appointment of a clinical risk manager in all public and private accredited healthcare facilities [5]. By linking professional liability to adherence to national guidelines validated by the National Institute of Health via the Italian National Center for Clinical Excellence (CNEC), the law created an unprecedented convergence between regulatory compliance and evidence-based practice [7]. A national survey conducted five years after the law’s enactment found that awareness of the law was positively associated with adverse event knowledge and checklist adoption among healthcare workers, though overall awareness remained inadequate [8]. A subsequent evaluation demonstrated that the law had not been fully implemented, with the CRM role remaining weak in most healthcare facilities and 72% of CRMs lacking regular interaction with regional centers for patient safety [9].

In parallel with the mandatory accreditation system, Italian healthcare organizations have increasingly adopted voluntary quality frameworks. International Organization for Standardization (ISO) 9001:2015, the most widely recognized quality management system (QMS) standard, has been implemented in various Italian healthcare settings, from angiology units in university hospitals to clinical trial centers (CTCs) nationwide [10,11]. The Joint Commission International (JCI) accreditation program, introduced in Italy in 2001 through a partnership with Progea Servizi, has been achieved by approximately 28 Italian organizations [12]. These include highly specialized centers such as IRCCS Istituto Clinico Humanitas in Milan (first accredited in 2002) and Ospedale Pediatrico Bambino Gesù in Rome (first accredited in 2006, achieving its seventh Gold Seal in 2024 as an Academic Medical Center) [13,14].

The publication of ISO 7101:2023 in October 2023 represents a potentially transformative development. Developed by ISO Technical Committee 304 (Healthcare Organization Management) with contributions from 30 nations under the leadership of the United States, this standard is the first international consensus standard specifically designed for healthcare quality management [15]. Unlike ISO 9001, a generic process quality standard applicable across industries, ISO 7101 prescribes requirements tailored to the healthcare sector, centered on people-centered care, the Plan-Do-Study-Act (PDSA) improvement cycle, and a 10-clause structure addressing timely, safe, effective, efficient, equitable, and people-centered care [15]. As of December 2024, while one international empirical study has evaluated a specific subclause of ISO 7101, no peer-reviewed evidence has been published on its implementation in Italian healthcare settings, highlighting this standard as an emerging area requiring scholarly attention.

The SSN is also subject to regulatory requirements established by Ministerial Decree 70/2015, which defined qualitative, structural, and quantitative hospital standards, and Ministerial Decree 77/2022, which redesigned territorial healthcare in the context of the National Recovery and Resilience Plan (PNRR) [16,17]. The Programma Nazionale Esiti (PNE), administered by Agenzia Nazionale per i Servizi Sanitari Regionali (AGENAS), monitors healthcare quality through 218 indicators applied to 1,117 facilities, consistently documenting a persistent North-South divide in healthcare quality [18]. This pattern is mirrored by the heterogeneous adoption of standardized clinical terminologies across regions [19].

Despite this complex and evolving quality ecosystem, no scoping review has comprehensively mapped the adoption, integration, and interaction of mandatory accreditation, ISO standards, JCI accreditation, and ISO 7101 within the Italian healthcare system. This review addresses the gap by systematically mapping the available evidence on quality management frameworks in Italian healthcare during the period 2016-2024, with particular attention to the impact of Law 24/2017, the role of voluntary ISO and JCI standards, and the emerging potential of ISO 7101.

The research question was formulated using the Population, Concept, Context (PCC) framework. The population comprises healthcare organizations and professionals operating within the Italian SSN. The concept encompasses the adoption, integration, and outcomes of quality management frameworks, including mandatory institutional accreditation, ISO 9001:2015, JCI accreditation, ISO 7101:2023, and the regulatory impact of Law 24/2017. The context includes Italian healthcare facilities, hospitals, IRCCS, clinical trial centers, and territorial care structures, during the period January 2016-December 2024.

## Review

Methods

Study Design

This scoping review was conducted in accordance with the updated JBI methodological guidance for scoping reviews and reported in accordance with the Preferred Reporting Items for Systematic Reviews and Meta-Analyses Extension for Scoping Reviews (PRISMA-ScR) checklist [20,21]. The methodological framework was based on the approach proposed by Arksey and O’Malley [22], as further refined by Levac et al. [23]. The review protocol was registered on the Open Science Framework (OSF) to enhance transparency and reproducibility [24]. In accordance with JBI guidance, no formal quality appraisal of included sources was performed, as the purpose of a scoping review is to map the extent and nature of available evidence rather than to produce a weighted synthesis of findings [20].

Search Strategy

A systematic search was performed in two electronic databases, PubMed/MEDLINE and Scopus, covering the period from January 1, 2016, to December 31, 2024. The search strategy was developed in consultation with a biomedical librarian to ensure comprehensiveness and reproducibility. The start date (2016) was selected to capture the period immediately preceding the enactment of Law 24/2017, representing the principal regulatory shift in Italian healthcare quality governance during the study period. The choice of PubMed and Scopus was motivated by their complementarity: PubMed for its comprehensive biomedical literature coverage with controlled MeSH vocabulary, and Scopus for its broader interdisciplinary indexing of European healthcare management journals. Table 1 presents the Boolean search strings used in each database.

**Table 1:** Boolean search strings used in PubMed and Scopus

Database	Search string
PubMed	("Total Quality Management"[MeSH] OR "ISO 9001" OR "accreditation"[tiab] OR "quality management system"[tiab] OR "ISO 7101" OR "JCI" OR "Joint Commission"[tiab] OR "clinical risk management"[tiab]) AND ("Italy"[MeSH] OR "Italian"[tiab] OR "Italy"[tiab]) AND ("Hospitals"[MeSH] OR "healthcare"[tiab] OR "health care"[tiab] OR "National Health Service"[tiab]) Filters: 2016-2024; English, Italian
Scopus	TITLE-ABS-KEY(("quality management" OR "ISO 9001" OR "accreditation" OR "JCI" OR "Joint Commission" OR "ISO 7101" OR "clinical risk management") AND ("Italy" OR "Italian") AND ("hospital" OR "healthcare" OR "health service")) AND PUBYEAR >2015 AND PUBYEAR <2025

The database search was supplemented by a targeted grey literature search of: (a) the AGENAS portal and PNE; (b) the Italian Ministry of Health technical and regulatory documentation section; (c) the official ISO website for ISO 7101 documentation; (d) the JCI-accredited organizations database; and (e) the Gazzetta Ufficiale della Repubblica Italiana for relevant legislation. Reference lists of included articles were also hand-searched (snowball searching) to identify additional relevant sources.

Eligibility Criteria

Sources were included if they were empirical studies of any design, reviews, policy analyses, or institutional reports addressing QMSs, accreditation, or clinical risk management in Italian healthcare. Documents analyzing ISO 9001, ISO 7101, JCI accreditation, or institutional accreditation in these settings were also included, as were international studies with direct transferability to the Italian context, such as systematic reviews of accreditation or ISO in healthcare. Legislative acts and normative documents defining the Italian quality governance framework were eligible. All included sources were published between January 2016 and December 2024 and were available in English or Italian. Sources were excluded if they focused exclusively on non-healthcare sectors, had no relevance to the Italian SSN or transferability to the Italian context, were editorials, letters, commentaries, or conference abstracts lacking full text, or addressed only clinical outcomes without reference to QMSs or frameworks.

Source Selection

The selection process was conducted independently by two reviewers (GF, CM) in three sequential phases: (1) title and abstract screening for relevance to the PCC question; (2) full-text review of potentially eligible sources; and (3) final application of inclusion/exclusion criteria with consensus. Disagreements were resolved by discussion or by involving a third reviewer (FM). The selection process was documented using a PRISMA 2020 flow diagram for scoping reviews [25].

Data Extraction (Charting)

For each included source, data were extracted into a predefined charting table following JBI recommendations, comprising: bibliographic reference, authors and year, country/region, study design, setting, population/sample, quality framework(s) examined, quality indicators or outcomes assessed, and principal findings [26]. The charting table was piloted on five sources and subsequently refined for consistency and completeness. The complete charting is presented in Table 2.

**Table 2:** Charting table: sources of evidence included (n=26) Table 2 presents the charting table of the 26 sources of evidence included in the scoping review. Reference numbers correspond to the bibliography and follow the order of first citation appearance in the text. References [12,19,20-26] are excluded from the charting table, as they underpin the review’s methodological structure (JBI guidance, PRISMA-ScR, Arksey-O’Malley, Levac et al., OSF protocol registration, Page et al., Pollock et al.), the JCI organizations registry, and a prior narrative review, rather than constituting primary sources of evidence. Consequently, reference numbering in this table is not consecutive (26 rows; references [1-34], with nine excluded). Data were extracted following JBI recommendations [26] into eight predefined domains: bibliographic reference, region/country, study design, setting, population/sample, quality framework(s) examined, quality indicators assessed, and key findings. CRM: clinical risk management; HCW: healthcare worker; HL: health literacy; IRCCS: Istituto di Ricovero e Cura a Carattere Scientifico; PNE: Programma Nazionale Esiti; SSN: Servizio Sanitario Nazionale; AMI: acute myocardial infarction; PDSA: Plan-Do-Study-Act; SNLG: National Guidelines System; CNEC: National Center for Clinical Excellence; QMS: quality management system; CTC: clinical trial centers; PNRR: National Recovery and Resilience Plan; HF: hip fracture; ISO: International Organization for Standardization; JCI: Joint Commission International; EU: European Union; AGENAS: Agenzia Nazionale per i Servizi Sanitari Regionali; EFQM: European Foundation for Quality Management; PRISMA-ScR: Preferred Reporting Items for Systematic Reviews and Meta-Analyses Extension for Scoping Reviews

Ref.	Author, year	Region	Design	Setting	Sample	Framework(s)	Indicators	Key findings
[1]	WHO (2006)	International	Policy framework	Health systems	Global	WHO quality dimensions	Efficacy, efficiency, accessibility, equity, safety	Six quality dimensions framework: effective, efficient, accessible, acceptable, equitable, safe
[2]	Shaw (2000)	EU (multi-country)	Cross-national study	European hospitals	ExPeRT project	Accreditation, ISO, EFQM	External quality mechanisms	Four main external assessment models; ISO certification is the most widespread; accreditation shows mixed evidence
[3]	Di Stanislao, Liva (2014)	Italy (national)	Policy analysis	SSN	Regulatory framework	Accreditamento istituzionale	Process quality, compliance	Accreditation system fragmented across 20 regions; heterogeneous implementation; need for national convergence
[4]	Legislative Decree 502/1992	Italy (national)	Legislation	SSN	All healthcare structures	Accreditamento istituzionale	Authorization, accreditation, contracts	Three-tier system: authorization, institutional accreditation, contractual agreements
[5]	Law 24/2017	Italy (national)	Legislation	SSN	All HCWs and facilities	CRM	Patient safety, liability	Mandatory CRM in all facilities; patient safety as a fundamental right; guideline-based liability framework
[6]	Bellandi et al. (2017)	Italy	Commentary/policy	SSN	National regulatory	Law 24/2017	Patient safety as a right	Italy, the first country to recognize patient safety as a fundamental right via law
[7]	Zerbo et al. (2020)	Italy	Policy/legal analysis	SSN	National guidelines (SNLG)	Gelli-Bianco Law; CNEC	Guideline adherence, liability	Adherence to national guidelines reduces malpractice claims; few guidelines published in SNLG to date
[8]	Albano et al. (2022)	Italy (national)	Cross-sectional survey	Multi-hospital	445 HCWs	Gelli-Bianco Law survey	Knowledge, sentinel events, checklists	Reading the law positively associated with adverse event knowledge and checklist adoption; education still inadequate
[9]	Candido et al. (2023)	Italy (national)	Cross-sectional survey	SSN	11 Regional Centers; 68 CRMs	Law 24/2017 implementation	Risk management, CRM activities	Law not fully implemented 5 years later; CRM role weak in most facilities; 72% CRMs lack interaction with regional centers
[10]	Avruscio et al. (2022)	Veneto	Case study (pre-post)	1 University Hospital (Angiology unit)	Angiology unit staff+patients	ISO 9001:2015 QMS	Patient safety, communication, clinical leadership	QMS improved patient safety systems, data transmission, clinical leadership; 14-month implementation
[11]	Franchina et al. (2023)	Italy (national)	Cross-sectional survey	CTC	88 respondents from Italian CTCs	ISO 9001:2015 survey	Benefits, barriers of QMS	73.3% report continual improvement; 60.7% risk management; main barrier: increased logistical activities (40.9%)
[13]	Humanitas (2002)-ongoing	Milan, Italy	Institutional report	1 IRCCS hospital	JCI standards	JCI hospital program	Quality benchmarking	First Italian JCI-accredited organization (2002); continuous triennial renewal
[14]	Bambino Gesù (2024)	Rome, Italy	Institutional report	1 pediatric hospital	1,200+ measurable elements	JCI academic medical center	Quality, safety, governance	7^th^ Gold Seal; first Italian pediatric JCI (2006); academic medical center since 2015
[15]	ISO (2023)	International	Standard (normative)	Any healthcare organization	Global applicability	ISO 7101:2023	People-centered care, PDSA cycle	First international healthcare QMS standard; 10 clauses; developed by ISO TC 304 (30 nations)
[16]	DM 70/2015	Italy (national)	Regulation	SSN	Hospital standards	Structural/organizational standards	Volume/outcome thresholds	Defined minimum qualitative, structural, and quantitative standards for hospital care
[17]	DM 77/2022	Italy (national)	Regulation	SSN	Territorial care standards	Community care models	Reorganization of primary care	PNRR-driven redesign of territorial healthcare; community houses, family nurses
[18]	AGENAS PNE (2025)	Italy (national)	Institutional report	1117 facilities	218 indicators	PNE monitoring system	Quality, appropriateness, safety	Persistent North-South divide; 15 hospitals achieve excellence (14 in Centre-North)
[27]	Colais et al.(2022)	Italy (national)	Retrospective cohort	SSN (national)	184 indicators; 2015-2020	PNE outcome evaluation	Hip fracture, AMI mortality, caesarean rates	HF surgery ≤2 days: 31.3%→64.6%; AMI 30-day mortality: 10.4%→8.3%; public reporting drives improvement
[28]	Fiore et al. (2024)	Italy (national)	Retrospective cohort	288 poorest hospitals	8 mortality indicators; 2016-2021	PNE impact evaluation	30-day mortality (8 conditions)	51% of poorest hospitals improved; Southern location was the strongest negative predictor of improvement
[29]	Tozzo et al. (2018)	Veneto	Descriptive study	1 University hospital	Clinical ethics committee	ISO 9001:2015	Quality, safety in ethics consultation	ISO certification improved standardization and transparency of clinical ethics processes
[30]	McCaskill et al. (2025)	Spain	Quasi-experimental (pre-post)	1 University hospital	Specialty consultations	ISO 7101:2023 (subclause 8.10.5)	Health literacy, quality of care perceived	First empirical ISO 7101 study; HL scores 66.35→76.29 (p<.001 d quality perception improved)
[31]	Alkhenizan, Shaw (2011)	International	Systematic review	Hospitals	26 studies	Accreditation programs	Quality of care measures	Accreditation associated with improved quality in most studies; evidence heterogeneous
[32]	Greenfield, Braithwaite (2008)	International	Systematic review	Health sector	66 studies	Accreditation programs	Clinical and organizational performance	Accreditation promotes organizational change; limited evidence on clinical outcomes
[33]	Pomey et al. (2010)	Canada	Mixed methods	Healthcare organizations	Multi-site	Accreditation process	Organizational change	Accreditation stimulates change through self-assessment and external survey
[34]	Braithwaite et al. (2010)	Australia	Blinded stratified study	Hospitals	19 hospitals	Accreditation vs. performance	Clinical and organizational indicators	Accreditation scores positively correlated with clinical indicators in some areas
[35]	Yousefinezhadi et al. (2015)	International	Systematic review	Hospitals	7 studies (ISO/EFQM)	ISO 9001; EFQM	Hospital performance	Limited but positive evidence; both models may improve hospital performance

Synthesis and Presentation of Results

Consistent with JBI methodology for scoping reviews, results were synthesized using a narrative approach, organized by emergent themes: (a) mandatory institutional accreditation; (b) the regulatory impact of Law 24/2017; (c) ISO 9001:2015 adoption; (d) JCI accreditation; (e) the emerging role of ISO 7101:2023; and (f) knowledge gaps and implications for healthcare management.

Results

Source Selection

The database search yielded 651 records (PubMed: n=334; Scopus: n=317). After removal of duplicates (n=99), 552 records were screened based on title and abstract. Of these, 497 were excluded for irrelevance to the PCC question. The remaining 55 records underwent full-text assessment, with 38 excluded for the following reasons: no focus on quality management frameworks (n=15), non-Italian context without transferability (n=10), no organizational or system-level analysis (n=6), full text unavailable (n=4), and editorials or commentaries (n=3). Seventeen sources were included from the database pathway.

**Figure 1:**
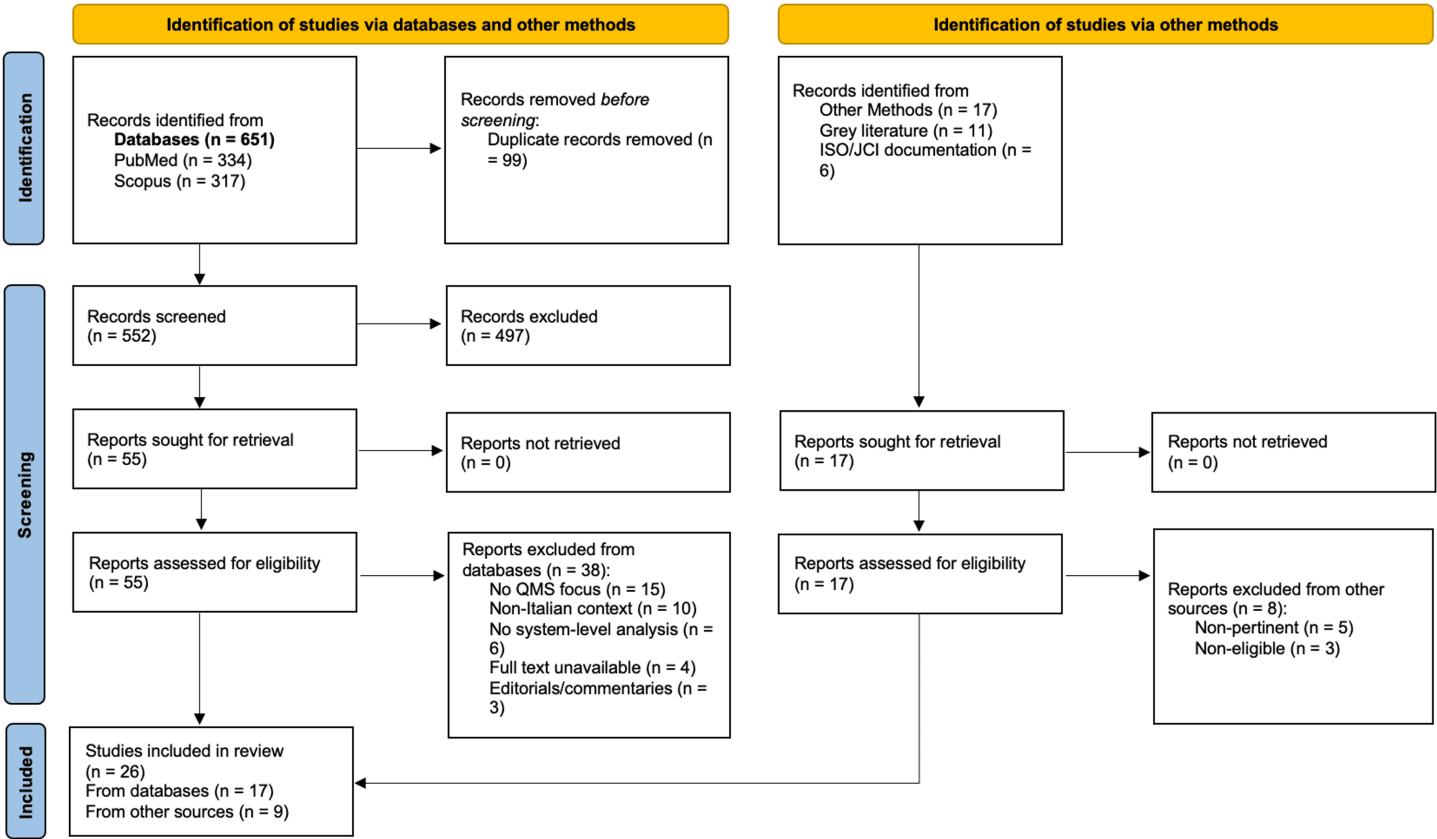
PRISMA 2020 flow diagram adapted for scoping review A PRISMA 2020 flow diagram adapted for scoping reviews, illustrating the study selection process. A systematic search of PubMed/MEDLINE and Scopus (January 2016-December 2024) yielded 651 records. After duplicate removal (n=99) and title/abstract screening (n=552), 55 reports were sought for retrieval from the database pathway and assessed for eligibility, with 38 excluded for the following reasons: no focus on QMSs (n=15), non-Italian context without transferability (n=10), no organizational or system-level analysis (n=6), full-text unavailable (n=4), and editorials or commentaries (n=3). An additional 17 records were identified through grey literature search (n=11) and ISO/JCI documentation review (n=6), of which eight were excluded for lack of relevance (n=5) or non-eligibility (n=3). After application of inclusion/exclusion criteria, 26 sources of evidence were included (17 from databases and 9 from other sources). Adapted from Page et al. [25] and reported in accordance with the PRISMA-ScR [21]. PCC: Population, Concept, Context; QMS: quality management systems; PRISMA-ScR: Preferred Reporting Items for Systematic Reviews and Meta-Analyses Extension for Scoping Reviews

Grey literature and supplementary searches identified 17 additional records (legislative acts, institutional reports, ISO documentation, and one international empirical study identified via snowball searching), of which eight were excluded due to lack of relevance (n=5) or non-eligibility (n=3), and nine were included. In total, 26 sources of evidence were included in the scoping review (17 from databases and nine from other sources). The selection process is illustrated in Figure 1. The complete charting table of included sources is presented in Table 2.

Charting Table of Included Sources

Table 2 presents the charting table of the 26 included sources of evidence. Methodological framework references, the narrative review, and the JCI organizations registry are excluded from the charting table, as they underpin the review’s structure rather than constituting primary sources of evidence.

Mandatory Institutional Accreditation and National Outcome Monitoring

The Italian institutional accreditation system was established by Legislative Decree 502/1992, which introduced a three-tier regulatory architecture: authorization to operate, institutional accreditation, and contractual agreements with local health authorities [3,4]. Di Stanislao and Liva documented that this system, while sharing a common national legislative foundation, is implemented heterogeneously across Italy’s 20 regions, each defining its own additional quality requirements beyond the minimum standards set by the 1997 Presidential Decree [3]. This regional fragmentation creates substantially different quality thresholds across the national territory, undermining the comparability of accredited structures and perpetuating disparities in healthcare quality [3]. Notably, no peer-reviewed empirical study published during the review period (2016-2024) evaluated the effectiveness of the accreditation process itself as a driver of quality improvement in Italian healthcare, highlighting a significant evidence gap.

The primary empirical mechanism for monitoring quality outcomes across accredited Italian facilities is the AGENAS PNE. The PNE 2025 report, which monitors 1117 facilities through 218 indicators, confirmed a persistent North-South divide, with 14 of 15 hospitals achieving excellence located in the Centre-North [18]. Colais et al. analyzed 184 PNE indicators over the 2015-2020 period and documented measurable national improvements: the proportion of hip fracture surgery within two days increased from 31.3% to 64.6%, and 30-day mortality after acute myocardial infarction decreased from 10.4% to 8.3%, with public reporting identified as a key driver of these improvements [27]. Fiore et al. evaluated the impact of the PNE on the 288 hospitals with the poorest performance in 2016, finding that 51.0% showed improvement by 2021. However, location in Southern Italy was the strongest negative predictor of improvement, confirming that the accreditation and monitoring framework yields uneven results across the national territory [28].

Law 24/2017 and CRM

Law 24/2017 (Gelli-Bianco) represents the most significant legislative intervention in Italian healthcare quality governance during the study period. By recognizing patient safety as a fundamental right and mandating the establishment of CRM structures in all healthcare facilities, the law created an unprecedented convergence between regulatory compliance and evidence-based practice [5,6]. Zerbo et al. analyzed the law’s linkage of professional liability to adherence to national guidelines validated by the CNEC, concluding that this mechanism has the potential to reduce defensive medicine, although its implementation has been hampered by the slow publication of guidelines in the National Guidelines System (SNLG) [7]. Albano et al. surveyed 445 healthcare workers across Italy and found that awareness of the law was significantly and positively associated with knowledge of adverse events (p<0.05), sentinel event communication, and checklist adoption. However, overall awareness remained inadequate, with only a minority of respondents demonstrating comprehensive knowledge of its provisions [8]. Candido G et al. evaluated the implementation five years post-enactment, surveying 11 regional centers and 68 CRMs. They found that the CRM role remains weak in most facilities, regional centers operate with an average of only two dedicated staff members, and 72% of CRMs lack regular interaction with these centers [9].

ISO 9001:2015 Adoption in Italian Healthcare

ISO 9001:2015 emerged as the most studied voluntary quality framework in Italian healthcare during the review period. Avruscio et al. described a 14-month implementation of an ISO 9001:2015-compliant QMS in the Angiology Unit of the University Hospital of Padua. The implementation was structured in five operational phases encompassing clinical management, clinical practice, safety, and patient-centeredness, and the study documented improvements in patient safety systems, communication protocols, and data transmission accuracy [10]. Franchina et al. conducted a national survey of 88 professionals from Italian CTCs, finding that the most frequently reported benefits of adoption of ISO 9001 QMS were continual improvement and improved process quality (73.3%), assurance of corrective actions (63.6%), planning of internal audits (60.2%), and a risk management approach (60.7%). The primary barriers included increased logistical and organizational activities (40.9%) and insufficient training on quality programs (29.5%) [11]. Tozzo et al. documented the application of ISO 9001 certification to clinical ethics consultation services at the University of Padua, demonstrating that formalization through the QMS improved the standardization, transparency, and accountability of ethics support services [29].

JCI Accreditation in Italy

JCI accreditation in Italy remains concentrated among highly specialized centers with significant organizational and financial resources. As of December 2024, approximately 28 Italian healthcare organizations hold active JCI accreditation, with Progea Servizi serving as the exclusive JCI partner in Italy since 2001. IRCCS Istituto Clinico Humanitas in Milan was the first Italian organization to achieve JCI accreditation in December 2002, maintaining continuous triennial accreditation renewal as an Academic Medical Center [13]. The Ospedale Pediatrico Bambino Gesù, the first Italian pediatric hospital accredited by JCI in 2006, received its seventh consecutive Gold Seal in 2024, following assessment against over 1200 measurable elements encompassing patient-centered care, infection control, hospital governance, and clinical research [14]. The concentration of JCI-accredited organizations in Northern and Central Italy mirrors the broader quality divide documented by the PNE [18,28]. It should be noted that the evidence on JCI accreditation in Italy is based entirely on institutional reports and registry data; no peer-reviewed empirical study conducted in Italian JCI-accredited facilities was identified during the review period, representing a notable gap in the evidence base.

ISO 7101:2023: A Healthcare-Specific Quality Standard

ISO 7101:2023, published on October 3, 2023, by ISO Technical Committee 304 with contributions from 30 nations, represents the first international standard specifically designed for healthcare quality management [15]. The standard prescribes requirements organized in 10 clauses aligned with ISO Annex SL while incorporating healthcare-specific content: people-centered care grounded in respect, compassion, co-production, equity, and dignity; the Plan-Do-Study-Act (PDSA) cycle for continuous quality improvement; and applicability to any healthcare organization regardless of size, structure, or location [15]. Unlike ISO 9001, a generic process standard applicable across industries, ISO 7101 addresses clinical, ethical, and organizational dimensions simultaneously, positioning it as a potential bridge between process-oriented quality management and outcome-oriented accreditation models.

The first peer-reviewed empirical study on ISO 7101 implementation was published by McCaskill et al. in December 2024. This quasi-experimental study, conducted over 12 months in specialty consultations at a Spanish university hospital, evaluated the implementation of ISO 7101 subclause 8.10.5 (health literacy). Nurses were trained and used a standardized health literacy checklist aligned with ISO 7101 requirements. Mean general health literacy scores increased significantly from 66.35 to 76.29, and mean perceived quality of care scores improved from 3.87 to 3.99, with both differences reaching statistical significance (p<0.001) and large effect sizes (d≥0.8). Although conducted outside Italy, this study provides the first empirical evidence that ISO 7101 implementation can produce measurable improvements in patient outcomes, providing a methodological model transferable to the Italian SSN context. No Italian study on ISO 7101 implementation was identified during the review period, confirming this as a priority area for future research.

Discussion

Synthesis of Principal Findings

This scoping review mapped 26 sources of evidence on the quality management landscape in Italian healthcare during 2016-2024. The findings reveal a system characterized by stratified governance without integration: mandatory institutional accreditation provides the regulatory floor, Law 24/2017 adds a patient safety mandate with legal consequences, ISO 9001:2015 offers voluntary process optimization, JCI provides international benchmarking for highly specialized institutions, the PNE monitors outcomes nationally, and ISO 7101:2023 introduces a healthcare-specific, people-centered paradigm that has not yet been implemented [3,5,7,10,11,13-15,18]. Each framework addresses a distinct quality dimension, yet no formal mechanism integrates them at the national or regional level. This creates a paradox: Italian healthcare organizations operate within one of the most complex quality governance architectures in Europe, yet the evidence suggests that this complexity does not translate into uniformly high-quality care, as demonstrated by the persistent North-South divide and the incomplete implementation of the law intended to unify safety culture [9,18].

From Regulatory Compliance to Value-Based Quality: A Four-Phase Trajectory

The evidence supports a conceptual model of Italian healthcare quality governance evolving through four sequential phases. Phase 1 (1992-2016) was defined by structural compliance: Legislative Decree 502/1992 established minimum standards for authorization and accreditation, delegated to Italy’s 20 regional systems with heterogeneous implementation [3,4]. Phase 2 (2017-ongoing) introduced mandatory safety culture: Law 24/2017 elevated patient safety to a constitutional right within the legal framework, mandated CRM structures, and linked professional liability to guideline adherence [5-7]. Phase 3 (concurrent) encompasses voluntary process excellence: ISO 9001:2015 adoption, documented in Italian settings with measurable benefits, including 73.3% of respondents reporting improved processes and demonstrable gains in patient safety communication, and JCI accreditation for institutions pursuing international benchmarking [10,11,13,14]. Phase 4 (emerging) represents value-based, people-centered quality: ISO 7101:2023, with its healthcare-specific PDSA framework and emphasis on equity, dignity, and co-production, offers a potential unifying paradigm that could align the safety imperatives of Law 24/2017 with the process rigor of ISO 9001 and the outcome orientation of JCI [15]. This four-phase model is not merely descriptive; it provides Italian healthcare managers with a strategic framework for sequencing quality investments based on organizational maturity.

The Implementation Gap: Why Legislation Is Necessary but Insufficient

A critical finding of this review is the divergence between legislative ambition and operational reality. Law 24/2017 mandated the appointment of CRMs in every public and private accredited facility, yet five years later, Candido et al. found that most CRM roles remain weak, regional centers have fewer than two dedicated staff members on average, and less than 20% of regional centers publish data on litigation and sentinel events for the public [9]. Albano et al. documented that, although reading the law is associated with improved safety knowledge, overall awareness of its provisions remains inadequate among healthcare workers [8]. Zerbo et al. further showed that only a limited number of national clinical guidelines have been published in the SNLG, undermining the law’s mechanism of linking liability to guideline adherence [7]. At the outcome level, Colais et al. [27] demonstrated that public reporting of quality indicators drives measurable improvement nationally, yet Fiore et al. showed that this improvement is geographically asymmetric: among the 288 poorest-performing hospitals, location in Southern Italy was the strongest negative predictor of quality improvement, even after adjusting for admission volume and baseline performance [28]. These findings converge on a single conclusion: legislative mandates and monitoring systems create the preconditions for quality culture transformation but are insufficient without sustained investment in human resources, training infrastructure, and accountability mechanisms, particularly in Southern regions where the infrastructure gap is widest [18,28].

ISO 7101:2023 as a Strategic Opportunity for the Italian SSN

The first empirical evidence on ISO 7101 implementation, published by McCaskill et al. at the close of the review period, demonstrated that implementation of subclause 8.10.5 (health literacy) produced statistically significant improvements in patient health literacy scores and perceived quality of care in a Spanish hospital setting [30]. While limited to a single subclause and a non-Italian context, this study provides proof of concept that ISO 7101 can translate into measurable patient outcomes. The standard’s architecture suggests several strategic advantages for the Italian SSN. First, its people-centered foundation, emphasizing respect, compassion, equity, and co-production, aligns directly with the patient safety philosophy embedded in Law 24/2017, potentially providing a structured methodology to operationalize the law’s aspirational principles [5,15]. Second, its PDSA cycle framework complements the risk management approach that Franchina et al. identified as one of the most valued benefits of ISO 9001 adoption (60.7% of respondents) [11,15]. Third, its 10-clause structure, aligned with ISO Annex SL, would facilitate integration with existing ISO 9001 certifications, reducing the “standard fatigue” that Franchina et al. documented as a significant barrier (40.9% citing increased logistical activities) [11].

We therefore propose that Italian healthcare policymakers consider ISO 7101:2023 as a candidate framework for the next phase of SSN quality governance, particularly in the context of PNRR-funded healthcare reorganization under DM 77/2022 [17]. Early adoption pilot programs in selected IRCCS or university hospitals, replicating the McCaskill et al. methodology across multiple ISO 7101 subclauses, could generate the comprehensive Italian implementation evidence currently lacking. In parallel, regional health authorities could explore alignment of ISO 7101 with existing accreditation requirements to create an integrated quality pathway.

Comparison With International Evidence

The Italian experience is consistent with the broader international literature. The WHO quality framework, which identifies six dimensions of quality care: effectiveness, efficiency, accessibility, acceptability, equity, and safety, provides the conceptual foundation that ISO 7101 operationalizes into a certifiable standard [1,15]. Shaw’s ExPeRT project documented the coexistence of multiple external quality mechanisms in European healthcare systems, with ISO certification being the most widespread [2]. Systematic reviews by Alkhenizan and Shaw [31] and Greenfield and Braithwaite [32] found that accreditation programs are generally associated with improved quality, although the evidence remains heterogeneous and mechanism-dependent. Pomey et al. [33] demonstrated that the accreditation process itself, through self-assessment and external survey, stimulates organizational change, while Braithwaite et al. found positive correlations between accreditation scores and clinical performance indicators in some areas [34]. Yousefinezhadi et al. reviewed the evidence on ISO 9001 and the EFQM model, concluding that both may improve hospital performance, although robust empirical evidence remains limited [35]. The Italian case adds a distinctive dimension to this discourse: the Gelli-Bianco Law’s integration of patient safety into the professional liability framework represents a mechanism not documented in other European systems, creating both a stronger incentive for quality adoption and a risk of defensive compliance rather than genuine quality culture [7,8].

Limitations

This scoping review has several limitations. First, the search was limited to two databases (PubMed/MEDLINE and Scopus); the inclusion of Cumulative Index to Nursing and Allied Health Literature (CINAHL), Web of Science, and Embase might have expanded the corpus, particularly for nursing and allied health perspectives. Second, the nature of scoping reviews precludes formal quality appraisal of included sources, limiting the ability to weight evidence by methodological rigor [20]. Third, the predominance of cross-sectional designs and institutional reports does not allow causal inferences regarding the effects of quality frameworks on clinical outcomes. Fourth, the empirical evidence on ISO 7101:2023 is limited to a single quasi-experimental study evaluating one subclause in a non-Italian setting; comprehensive implementation evidence across the standard’s full scope remains unavailable. Fifth, the persistent concentration of empirical evidence from Northern and Central Italy limits the generalizability of findings to the SSN as a whole and likely underestimates the extent of quality governance challenges in Southern regions.

## Conclusions

This scoping review demonstrates that the Italian healthcare quality landscape is undergoing a structural transition from a compliance-driven model rooted in Legislative Decree 502/1992 toward a multi-framework ecosystem integrating legislative mandates (Law 24/2017), voluntary process standards (ISO 9001:2015), international accreditation (JCI), and national outcome monitoring (AGENAS/PNE). Each framework addresses a distinct quality dimension: structural compliance, safety culture, process quality, and international benchmarking. However, these elements remain largely siloed, with no formal integration mechanism at the national or regional level. The publication of ISO 7101:2023 represents a strategic inflection point. As the first international standard specifically designed for healthcare quality management, and supported by preliminary empirical evidence, it offers a potential unifying paradigm aligning the safety imperatives of Italian legislation with the process rigor of ISO and the focus on patient-centered care increasingly demanded by patients and regulators alike.

Based on the evidence synthesized, five priorities emerge for future action: empirical pilot studies evaluating ISO 7101 adoption in Italian IRCCS or university hospitals with existing ISO 9001 certification; targeted investment in CRM training and regional center staffing to close the Law 24/2017 implementation gap; integration of ISO 7101 requirements with existing institutional accreditation criteria to create a unified quality pathway; multicenter studies investigating the quality governance gap in Southern Italian regions; and economic analyses of the return on investment of voluntary certification programs for SSN facilities.
